# Supplementary figures and images for: Identifying small molecule probes of ENTPD5 through high throughput screening
Source: PLoS One. 2019 Jun 26;14(6):e0210305. doi: 10.1371/journal.pone.0210305 (PMC6594577; doi:10.1371/journal.pone.0210305)

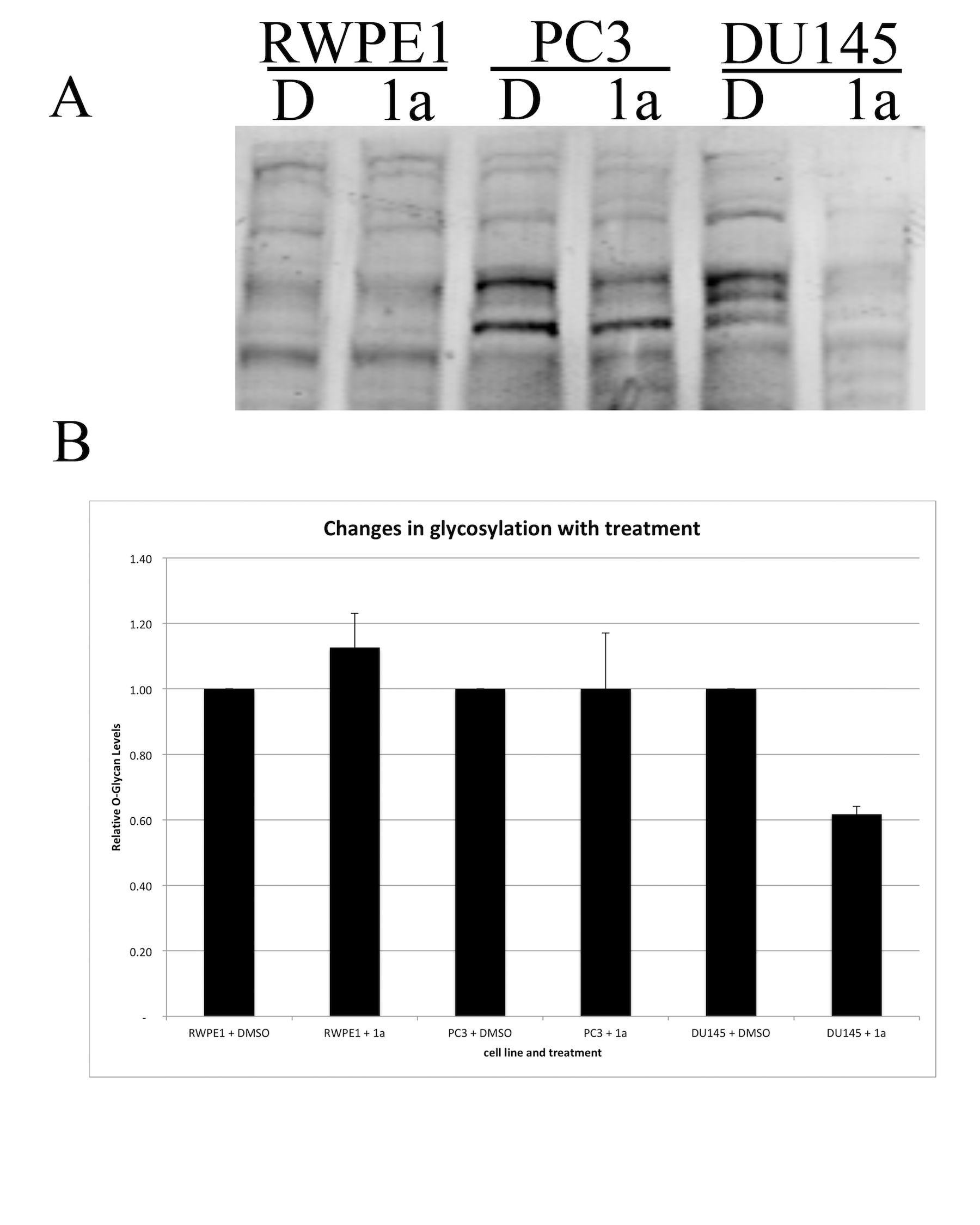

Supplement: S1 Fig — A) Levels of O-glycan in RWPE-1, PC3, and DU145 cells treated with 10 μM 1a or DMSO for 24h. Relative protein amounts were normalized to DMSO-treated cells from each cell line. B) Quantification of glycosylation normalized to DMSO treated cells and normalized to total protein. Error bars represent standard deviation between 2 experiments. (TIFF) [file pone.0210305.s001.tiff]

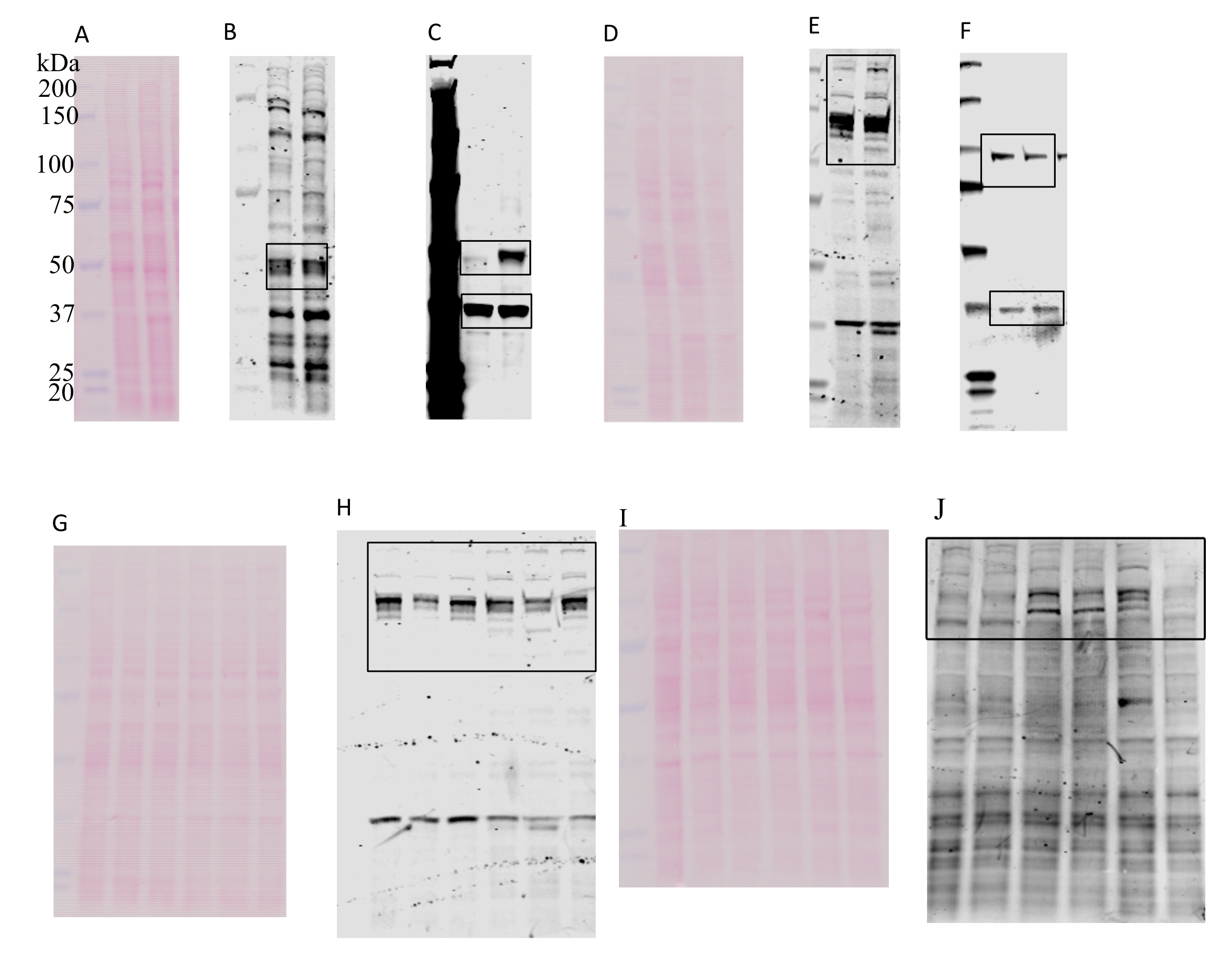

Supplement: S2 Fig — A) Ponceau for Fig 6A ENTPD5 & PTEN. B) Uncropped image for Fig 6A ENTPD5 on the 800 nm channel. C) Uncropped image for 6A PTEN (upper box) and GAPDH (lower box) in the 700 nm. D) Ponceau for Fig 6A O-Glycan. E) Uncropped image for Fig 6A O-Glycan on the 800 nm channel. F) Uncropped image for Fig 6A SP1 (upper box) and GAPDH (lower box) on the 700 nm channel. G) Ponceau for Fig 6B. H) Uncropped image for Fig 6B O-Glycan on the 800 nm channel. Boxes represent area cropped for figures in the manuscript. I) Ponceau for S1A Fig. J) Uncropped image for S1A Fig O-Glycan on the 800 nm channel. Boxes represent area cropped for figures in the manuscript. (TIF) [file pone.0210305.s002.tif]
